# Broader Validation of New Zealand Eating Behavior Questionnaire as Clinical Assessment Tool to Identify Actionable Eating Behavior Traits

**DOI:** 10.3390/nu17061049

**Published:** 2025-03-17

**Authors:** Ole Schmiedel, Melissa Ivey, Rinki Murphy

**Affiliations:** 1 Auckland Diabetes Centre, Te Toka Tumai Auckland, Auckland 1051, New Zealand; 2 Department of Medicine, Faculty of Medical and Health Sciences, University of Auckland, Auckland 1023, New Zealand; 3 Ivey Public Health Partners, LLC, Atlanta, GA 30308-1621, USA

**Keywords:** appetite, eating behavior, obesity, questionnaire, validation

## Abstract

**Background/Objectives**: The New Zealand Eating Behavior Questionnaire (NZ-EBQ) is a validated questionnaire that comprises three distinct scales that measure satiation at mealtimes, satiety in the post-eating period, and emotional eating behavior. This study evaluated the model validity of the NZ-EBQ across two additional samples of demographically diverse participants using confirmatory factor analysis. **Methods**: We compared the classification of the eating behavior (EB) type with that of the initial cohort used to develop the three-factor model. Two cohorts of 81 and 214 participants provided complete data sets for analysis. Cohort 1 was characterized by the use of more weight management medications, and participants in Cohort 2 were significantly heavier. Confirmatory factor analysis was performed using combined data from both cohorts to maximize the sample size. **Results**: Except for one item, all items demonstrated a factor loading consistent with the established three-factor model. After removing one item from the emotional eating scale, the model fit statistics did not change significantly. Participants were assigned to one of the three EB types based on their highest median score, and most could be classified into one of the three EB types, with only a few who could not be classified (Cohort 1:12.3%; Cohort 2:13.0%). The test-retest reliability performed in a subset of participants was comparable to that of the initial validation cohort. A significant positive correlation was found between BMI and the individual EB scores. **Conclusions**: The NZ-EBQ may serve as a screening tool for identifying actionable EB traits that help select targeted interventions based on EB, supporting precision medicine-based approaches.

## 1. Introduction

The global prevalence of obesity has rapidly increased in recent decades and is now one of the foremost issues facing healthcare providers [1]. Obesity is associated with reduced life expectancy and an increased risk of multiple health conditions, including type 2 diabetes, vascular disease, and several cancers [2]. Significant advances have been made in anti-obesity medications in recent years, with multiple agents currently available. These drugs are highly effective when administered in combination with lifestyle changes. Different weight loss medications can achieve a placebo-subtracted weight loss of 3 to 24% of body weight; however, a wide variation is observed between responders and non-responders [3]. Currently, few tools are available to predict the weight loss response of an individual to any given intervention [4]. Understanding which weight loss strategy would work best for any given individual would increase cost-effectiveness and improve health outcomes for individuals living with obesity [5]. One option would be selecting weight management medications based on observable dominant eating behaviors [6,7]. Eating behavior (EB), which describes the cognitive and emotional processes that motivate or drive a person to eat, is stimulated and inhibited by physiological control mechanisms that subsequently determine an individual’s energy intake. In addition, this system is impacted by several environmental, social, and cultural factors [8]. The underlying physiological mechanisms can be broadly distinguished between the homeostatic regulatory system, which includes hypothalamic control centers and hormonal and neuronal communication, and hedonistic pathways via the mesolimbic system [9]. Nevertheless, the significant overlap in pathways responsible for hedonistic and homeostatic eating behaviors makes a clear distinction difficult [10].

Several approaches and models exist that aim to identify and describe EB traits. Self-reported questionnaires are a widely studied approach. However, multiple questionnaires use overlapping constructs and theories to measure EB traits, with significant heterogeneity in cause-and-effect relationships [11]. One helpful concept is the satiety cascade, which describes appetite as a temporal continuum of satiation with meals (fullness upon eating) and satiety in the post-eating period (return of hunger after eating) at three levels of operation: behavioral pattern, peripheral metabolism, and brain activity, forming a psycho-biological system [12,13]. This theoretical framework is incorporated in the NZ-EBQ [14], where two dimensions relate to satiation and to satiety. The hedonistic pathway, which includes wanting, liking, and using food to deal with strong emotions, is represented separately on the emotional eating scale.

We recently presented the results of a validation study of the NZ-EBQ, in which the factor structure and construct validity were investigated in a large cohort of patients (n = 729), most of whom had diagnoses of type 2 diabetes and obesity, from an academic diabetes center. Exploratory factor analysis identified three main factors, or EB traits, which were confirmed using confirmatory factor analysis. These were (1) reduced satiation at mealtimes (feasters), (2) decreased satiety between meals (constant hunger), and (3) emotional eaters. The three-factor model was consistent across subgroups of sex, age, ethnicity, and educational level. The test-retest reliability measured in a sizable subset of the study population was moderate to high. The initial tool included 42 items, and after removing items with insufficient factor loading values, the final three-factor model contained 27 items, accounting for 95% of the observed variance. The three factors detected were comparable to those outlined by Acosta and Camilleri [7,15]. There was an overlap across several EB traits, indicating that most people have more than one motivator to eat, which aligns with the physiological mechanisms that regulate eating behavior. This is consistent with the findings of Acosta et al. [6], who found that 15% of participants had no discernible EB trait and 27% had two co-dominant EB traits.

Several EB questionnaires, including the widely used Three Factor Eating Questionnaire (TFEQ) [16], exist in different iterations since factor structure or item loading could not be replicated in subsequent cohorts, which resulted in items being removed and revised clustering of factors being proposed [17,18,19]. Few questionnaires to date have included people living with obesity who have received treatment with either medication or bariatric surgery, which limits the validity of using such questionnaires in clinical practice [19,20]. This study’s overall objective is to test the validity and reproducibility of the NZ-EBQ across different populations, including those with higher body weight, of different ethnicities, on weight loss medications, and in people without diabetes, especially as the initial study had a high number of patients with type 2 diabetes. To achieve this, we intentionally chose cohorts of patients awaiting or declining bariatric surgery, who were expected to be of different ethnicities and have higher body weight, and patients attending a private weight management clinic, where we expected more patients to be on weight loss medications. If item loading and factor structure can be replicated, the NZ-EBQ may be a reliable assessment tool across a wide range of potential users within the target population.

## 2. Materials and Methods

### 2.1. Instrumentation

Following the approach used in the initial validation study [14], participants were asked to complete the NZ-EBQ, a 42-item questionnaire that also included 14 items related to their demographics, current weight, previous weight loss attempts, and intention to lose weight. The questionnaire included two image-based questions, and two additional images were inserted as attention enhancers [21]. The image-based questions utilized multiple-choice responses, whereas most questionnaire items were formatted as visual analog scales (0 to 100) with anchor points at both ends. In addition, all participants were asked to self-assess their EB using a direct question to evaluate whether this response was comparable with the results obtained by the questionnaire.

### 2.2. Population and Samples

Two cohorts of patients with obesity from the Metro Auckland Area were approached for participation. The eligibility criteria included being willing and able to provide informed consent, age more than 18 years, and having the ability to read and understand English. There was no body mass index (BMI) cut-off or upper age limit for inclusion. We refer to the initial validation cohort (cohort 0) [14] as a reference cohort and benchmark when comparing model fit statistics, consistencies, and differences in the classification. The study protocol adhered to the Declaration of Helsinki and was approved by the Health and Disability Ethics Committee at Te Toka Tumai, Auckland (A+9296, 11 August 2021).

#### 2.2.1. Initial Cohort (Cohort 0)

The initial validation cohort, Cohort 0, was recruited from the Auckland Diabetes Centre, Auckland, New Zealand, a tertiary diabetes center. As such, most participants had a previous diagnosis of type II diabetes (74%) and reported being overweight (51%), although few were taking prescription weight loss medication at the time of the study (2%). The full demographic characteristics of this cohort have been published previously.

#### 2.2.2. Cohort 1

Participants in Cohort 1 were identified from a private weight management clinic in Auckland. These patients reflected the demographic characteristics of patients seen in medical bariatric clinics regarding ethnicity, education, age, and sex. Because this cohort consists of patients from a private clinic, these individuals are often of a higher socioeconomic status and may be more likely to use weight loss medication than those in Cohort 0, which may impact their responses to the questionnaire. Additionally, participants in Cohort 1 were expected to have a lower rate of diabetes than those in the initial cohort. Patients with diabetes often respond differently to weight management medications (e.g., GLP1 antagonists) than patients without diabetes [22,23], thus creating the need to validate the NZ-EBQ in this cohort.

#### 2.2.3. Cohort 2

The second cohort of participants was recruited from a database of patients referred by a publicly funded tertiary hospital in Auckland and awaiting or declined for bariatric surgery. These patients were expected to have a higher BMI, an increased number of obesity-related complications, and a different demographic composition than those in Cohorts 0 and 1.

### 2.3. Data Collection

Data collection for Cohort 1 was conducted between 30 January and 8 March 2022. The primary phase of data collection (Phase I) for Cohort 2 was conducted between 12 September and 1 December 2022. Participants in Cohort 2 were invited to complete the questionnaire again during Phase II to assess the test-retest reliability. Phase II was conducted between 2 December and 24 December 2022. First and last names were collected to match the responses in Phases I and II; however, all identifying information remained confidential. The questionnaire was administered electronically without supervision and was preceded by a participant information and consent form. The English-language questionnaire was accessible only to those who read the information document and consented to participate in the study.

### 2.4. Analysis

Descriptive statistics were calculated for the demographics, weight and weight loss characteristics, and self-assessed EB type of the participants in each cohort. Responses included in the analysis were compared to those excluded using Fisher’s exact test and independent sample *t*-tests. Chi-square, analysis of variance (ANOVA), and independent sample *t*-tests were used to compare the distribution of demographic characteristics among the three cohorts. Confirmatory factor analysis (CFA) was performed to validate the fit of the model previously reported in the literature by the authors. One item (“For how long can you go without food before you feel hungry”) was negatively worded and was therefore reverse coded. Because questionnaire items were determined to be non-normal, ranked variables were created for each item and used in CFA, and Spearman coefficients were used instead of the standard Pearson coefficients. The comparative fit index (CFI), Tucker-Lewis index (TLI), standardized root-mean-square residual (SRMR), and root-mean-square error of approximation (RMSEA) were calculated to assess the fit of the model [24], and Cronbach’s alpha (α) was used to evaluate the internal consistency of each factor. The highest median score observed across relevant items was used to categorize participants in each cohort into one of three main types of EB: emotional eaters, feasters with reduced satiation, or those with constant hunger with reduced satiety. Initially, the median scores for all items within each factor were calculated. The dominant EB type of each participant was determined using the highest median score among the EE, CH, and F scales. Subsequently, the scores were compared. For example, individuals who demonstrated a higher median score for emotional eating than for feasting or continuous hunger were classified as emotional eaters. Participants who achieved median scores that were equal across two or more factors were not classified as having a dominant EB type and were therefore excluded from further analysis. The median scores and interquartile ranges of the relevant factors for each EB type were reported. Pearson’s chi-square, Kruskal−Wallis, and Mann−Whitney U tests were employed to assess differences in EB classification and median scores among cohorts. The test-retest reliability of this classification was evaluated in Cohort 2 using the Kappa coefficient, with values between 0.61 and 0.80 considered ‘good’ and 0.81 and 1.00 ‘very good’ [25]. Similarly, intraclass correlation coefficients (ICC) were calculated for individual items and overall factors in the EB model to establish consistent measurements. A two-way mixed-effects model with absolute agreement was utilized, where ICC values between 0.50 and 0.75 indicated moderate reliability, 0.76–0.90 good reliability, and values exceeding 0.90 indicated excellent reliability [26]. Following the classification of participants’ EB type, we examined variations in demographic characteristics among individuals assigned to each EB type. Additionally, we investigated the association between EB type and BMI, self-classified EB type, and responses to image-based questions using Pearson’s chi-square test, Fisher’s exact test, and independent sample *t*-tests. All data analyses were performed using the Stata Statistical Software (Release 15, StataCorp, College Station, TX, USA). A *p* value of <0.05 was considered significant for all statistical tests.

## 3. Results

### 3.1. Participants

Of the 98 individuals who responded to the questionnaire as part of Cohort 1, 17 were excluded from the analysis because they did not consent (n = 2), did not meet the inclusion criteria (n = 5), or had some degree of item-level missing data (n = 10), including two who did not respond to any of the 42 items and eight who had partial incompleteness. After removing these responses, a final sample of n = 81 remained in Cohort 1.

From the second cohort, 244 individuals responded to the questionnaire during Phase I. Of these responses, 29 were excluded from the analysis because they did not consent (n = 4), did not meet the inclusion criteria (n = 6), or had some degree of item-level missing data (n = 19), including three who did not respond to any of the 42 items and 16 who had partial incompleteness, with at least 23% missing data. After removing these responses, a final sample of n = 215 remained in Cohort 2 for Phase I. Of these, 44 (20.5%) responded to the Phase II invitation to assess test-retest reliability. Participants completed the same 42-item questionnaire after a mean response interval of 11.4 weeks. After excluding participants with incomplete data, data were matched, and 38 participants were included in the test-retest reliability assessment.

### 3.2. Demographics

**Figure 1:**
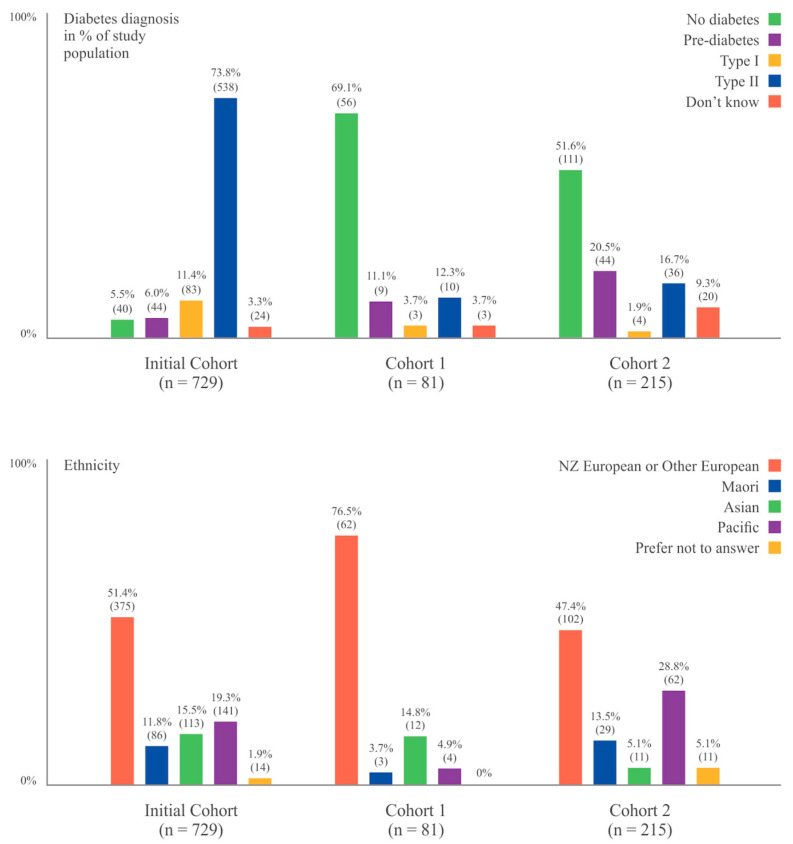
Prevalence of Diabetes and Ethnicity of Participants in the Two Cohorts Compared to Initial Cohort. The values are depicted as a percentage of participants within each cohort. The absolute number of participants is provided for reference.

When comparing the demographic distribution of participants in Cohorts 1 and 2 with those in the initial cohort (Cohort 0), statistically significant differences were observed in all demographic variables (Table 1). Cohorts 1 and 2 had a higher proportion of females (*χ*^2^ = 93.1; *p* = 0.000) and participants who reported that they were “very overweight” (*χ*^2^ = 252.5; *p* = 0.000), wanted to lose weight (*χ*^2^ = 51.7; *p* = 0.000), and were taking any registered weight loss medications at the time of questionnaire (*χ*^2^ = 163.8; *p* = 0.000) than the initial cohort. They also had a lower proportion of participants with a prior diagnosis of diabetes (*χ*^2^ = 474.7; *p* = 0.000) (Figure 1), were younger (*χ*^2^ = 186.1; *p* = 0.000), and reported a higher weight (*F* (2) = 152.96; *p* = 0.000) than previously described participants. Additionally, statistically significant differences between Cohorts 1 and 2 were observed for all demographic variables except for sex. Cohort 1 had a higher proportion of participants who reported taking weight loss medications (*χ*^2^ = 36.3; *p* = 0.000) and were older (*χ*^2^ = 13.3; *p* = 0.006) than those in Cohort 2. A higher proportion of respondents in Cohort 2 identified as Maori or Pacific ethnicity (*c*^2^ = 40.5; *p* = 0.000) (Figure 1) and reported that they were “very overweight” (*χ*^2^ = 42.0; *p* = 0.000) than those in Cohort 1. Most notably, Cohort 2 reported a mean weight of more than 37 kg heavier than that of Cohort 1 (*t* (288) = −7.37; *p* = 0.000). A comparison of responses that were included in the analysis of Cohorts 1 and 2 (n = 81 and 215, respectively) and those that were excluded based on full or partial missingness (n = 17 and 19) on responses to the survey items showed that there were no significant differences in demographics or weight loss characteristics between those with complete data and those with missing data.

### 3.3. Confirmatory Factor Analysis

**Figure 2:**
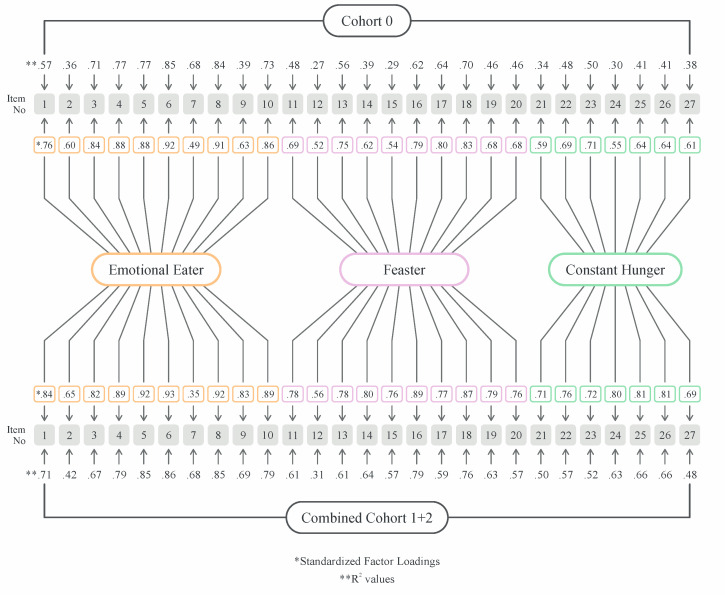
Graphical representation of the confirmatory factor analysis (CFA) of the three-factor model of eating behavior in the two cohorts compared to the initial cohort. * Standardized factor loading, ** *R*^2^ values.

Confirmatory factor analysis (CFA) was performed using the combined data from Cohorts 1 (n = 81) and 2 (n = 215) to maximize the sample size for determining the fit of the three-factor model previously reported in the literature by the authors. Figure 2 shows the standardized factor loadings and *R*^2^ values of each item in the combined cohort compared to the initial cohort (Cohort 0). Most items demonstrated a pattern of factor loadings consistent with the established three-factor model; however, one item did not load as expected.

Item 7, derived from the Yale Food Addiction Scale (YFAS) [27] (“Even though I know better, I continue eating in the same way when my eating causes me emotional or social problems”) cross-loaded onto both the emotional eater factor (0.35) and the constant hunger factor (0.52) and did not meet the 40-30-20 rule, suggesting that this item should be removed from the model. The authors previously reported that this item cross-loaded onto the emotional eater factor (0.69) and the constant hunger factor (0.41) but met the 40-30-20 rule and was therefore retained in the initial model. An ad hoc analysis was performed to assess the impact of removing item 7 on the model fit statistics and EB classification of participants. First, the fit statistics of the model with and without item 7 were compared for the present and initial cohorts (Table S1). In both models, the combined cohort demonstrated model fit statistics within the generally desired range (i.e., CFI and TLI > 0.90; SRMR and RMSEA ≤ 0.08) [24]. The model fit statistics for Cohort 0 did not significantly change when item 7 was removed, nor were there any meaningful changes in the internal reliability of the emotional eater factor from which the item was removed in either cohort. Therefore, the authors accepted the model fit statistics that supported the three-factor model.

Reclassifying the primary EB type of participants in the initial cohort using a new model that did not contain item 7 was also conducted to assess the impact of its exclusion. Table 2 shows the number and proportion of individuals assigned to each EB type using the original and new models. The number of participants classified as emotional eaters, on which scale factor item 7 was loaded in the original model, changed by only 1.0%. The number of participants identified as feasters or having constant hunger decreased by 3.4%, while the number of participants who could not be classified increased from 55 to 70. Overall, 31 changes in classification, representing 4.2% of the participants, were observed. When considering the median scores of the emotional eating factor among those classified as emotional eaters, the median increased slightly after the removal of item 7 (old model median = 65.2; 95% CI: 55.5–83.5 versus new model median = 67.1; 95% CI: 56.5–85.0), which may indicate that the new model is a better representation of this EB type. After considering these findings, item 7 was removed from the model.

### 3.4. Classifying Dominant Eating Behavior Type of Participants

Participants were assigned to one of three EB types based on the highest median score for the factors. For example, individuals with a higher median score on the feaster factor than on the emotional eater or constant hunger factor were classified as feaster. Using this strategy, 31 (38.3%) participants in Cohort 1 were classified as emotional eaters, 18 (22.2%) as feasters, and 22 (27.2%) as having a constant hunger (Table S2). Ten (12.3%) participants were tied on two or more factors and could not be classified using the highest median score. Among the participants in Cohort 2, 96 (44.6%) were classified as emotional eaters, 37 (17.2%) as feasters, and 54 (25.1%) as having a constant hunger. An additional 28 (13.0%) participants could not be classified as having a primary EB type. In both cohorts, the group classified as emotional eaters demonstrated the highest median score for their associated factors and the highest overall median score across all three factors (Table S3), as reported in the initial cohort study.

A significant difference was observed in the proportion of participants classified into each EB trait among cohorts (*χ*^2^ = 39.3; *p* = 0.000), (Table S4). The only demographic variation observed among participants assigned to the three EB types was sex. Females comprised a significantly larger proportion of emotional eaters than feasters or those experiencing constant hunger (*χ*^2^ = 19.2, *p* = 0.000). No other significant differences in demographic or weight loss characteristics were observed among the three EB traits.

### 3.5. Test-Retest Reliability

Using ICC values, most of the 26 survey items included in the new model displayed moderate (n = 14, 53.8%) or good (n = 9, 34.6%) test-retest reliability (Table 3). One item (“I know that I’m an emotional eater”) showed excellent reliability (0.92), whereas two items (“Do you feel a sensation of fullness during eating?” and “Whenever I have a food craving, I keep on thinking about eating until I actually eat the food.”) exhibited poor reliability (<0.50). Except for these few items, the findings were consistent with the test-retest reliability estimates obtained from the initial cohort, in which items displayed moderate to good reliability. Overall, the emotional eater scale demonstrated excellent reliability (0.92, 95% CI: 0.84–0.95). However, the feaster (0.75, 95% CI: 0.56–0.86) and constant hunger (0.65, 95% CI: 0.42–0.80) scales showed moderate reliability. Additionally, the classification of participants based on the dominant EB type in Phase I was compared with the classification of the same participants (n = 34) in Phase II (Table S5). A Kappa coefficient of 0.61 (76.7% agreement) indicated good reliability for this model and classification strategy.

### 3.6. Relationship Between Eating Behavior Trait and BMI

Because all participants reported at least some influence on their EB from the non-dominant EB traits, we calculated an aggregate EB score (i.e., the sum of the EE, F, and CH scores) for each participant. The aggregate score showed a significant correlation with BMI (r = 0.24, *p* = 0.000). Similar findings for each individual EB trait were observed (r = 0.18 (EE), 0.25 (F), 0.21 (CH), *p* < 0.002 (Figure 2).

### 3.7. Self-Assessed Eating Behavior of Participants

Participants in Cohorts 1 and 2 were also asked to self-categorize their EB type (Table S6). Over three-quarters (n = 56; 81.2%) of individuals who self-assessed as emotional eaters were classified as emotional eaters using median scores, and 63% (n = 35) of participants who were classified as feasters had self-reported as eating ‘large portion sizes’. However, fewer than one-third of participants classified as having CH self-identified as having ‘grazer or constant craving’. Among those in Cohort 2 who completed the survey during Phases I and II, the test-retest reliability of self-assessed EB was low (kappa = 0.45; 28.0% agreement).

### 3.8. Image-Based Questions

The “savouring the first bite” image-based question did not demonstrate a significant association with EB classification (*χ*^2^ = 17.9, *p* = 0.056). The second image-based question, which showed a sizzling steak, “visualizing taste and smell,” used Likert-style categorical responses, which were converted to numerical values. This image also showed no significant association, as it did not load onto any of the three factors (all factor loadings were < 0.21). Consequently, we removed the two image-based questions from our final tool. However, we retained the two attention-enhancer items.

## 4. Discussion

The NZ-EBQ was developed to identify distinct human EB traits that reflect the underlying physiological and neurobiological mechanisms consistent with the concepts described in the satiety cascade [28]. Here, we further evaluated the model validity and item factor loading in two additional cohorts drawn from diverse backgrounds that represent the target population of this questionnaire. Many widely used questionnaires have been validated in homogeneous populations of mainly healthy participants [19,29]; however, it is important that evaluation cohorts closely match the target population for the assessment tool. The mean weights of the participants in the three cohorts were 97.2, 102, and 139.3 kg, respectively, and we included sufficient participants from different ethnicities and those on weight loss medications. We were able to demonstrate that the NZ-EBQ is stable across different ethnic groups (Maori, Pacific people, NZ Europeans), knowing that eating behaviors, customary food practices, and cultural realities can vary significantly.

The previously validated NZ-EBQ had 27 items divided into three scales: ‘Constant Hunger’ (CH), which refers to reduced satiety or inter-meal levels of hunger; ‘Feasters’ (F), which describes a reduced or delayed satiation response that contributes to delayed meal termination and influences calories consumed; and “Emotional Eating” (EE), which describes overeating in response to a variety of negative emotions [30,31].

The original TFEQ, a 51-item tool that describes three aspects of eating behavior: cognitive restraint, disinhibition, and hunger, has been analyzed in various cohorts to assess its factor structure and construct validity. Several investigators discovered, however, that the original 51-item structure could not be fully replicated [16]; hence, the TFEQ-R18 with different scales was developed [17]. Nevertheless, the Dutch Eating Behaviour Questionnaire (DEBQ) [32] was initially validated in normal weight, overweight, and obese individuals, confirming a similar factor structure across different cohorts, comprising three scales: emotional, external, and restrained eating. Subsequently, several studies were able to replicate the three-factor structure [33,34]. Consistent with Van Strien’s approach, we investigated at this stage of the development process the internal consistency and factor loading of individual items in various cohorts encompassing individuals of different ethnicities, body weights, and education levels to create a reliable tool.

Our analysis revealed that the three-factor model remained stable across both cohorts; almost all items loaded onto the same factor, with similar standardized factor loadings and R values. Compared to the validation cohort, nine items were loaded on EE, ten on F, and seven on CH, with only one item (item 7) exhibiting cross-loading that did not meet the 40-30-20 rule, prompting us to investigate this item further. In the validation cohort, item 7, “Even though I know better, I continue eating in the same way when my eating causes me emotional or social problems”, also cross-loaded on EE and CH, with more substantial loading on the EE scale. In contrast, in Cohorts 1 and 2, item 7 loaded more strongly on CH, contradicting the initial findings. This item was derived from the YFAQ [27], which describes food addiction rather than emotional drivers of eating. After considering the model fit statistics and reviewing the wording of item 7 from a clinical perspective, the authors determined that this degree of change in EB classification was unacceptable and removed item 7 from the model. As we removed item 7, we needed to perform additional calculations to assess changes in the model fit statistics. Reassuringly, there was no change in internal reliability, and removing the item strengthened the EE scale from which it was removed. After removing item 7, the number of unassigned participants increased, reducing the number of falsely assigned participants and supporting the concept that several people cannot be classified with this tool as people may have multiple or co-dominant EB [6]. This observation clarifies that the tool needs to be used with other assessment methods [35] and performs best in those with a single dominant EB trait [10].

Caroleo et al. [36] conducted a study that categorized individuals into different groups based on their EB and genetic profiles. They found two main clusters: Cluster One consisted of individuals who exhibited hyperphagia and social eating, while Cluster Two consisted of individuals who showed increased emotional eating and had anxiety disorders as defined by the DSM-5. The research revealed highly significant differences, accompanied by substantial effect sizes, in scores obtained from various assessment tools, including the Binge Eating Scale [37], Beck’s Depression Inventory (BDI) [38], State and Trait Anxiety Disorder (TASI), and Eating Disorder Inventory-2 (EDI-2) [39], between the two cohorts. Our findings align with this observation, as the EE scale of the NZ-EBQ demonstrated the highest reliability and internal consistency scores, best reproducibility, and highest median score for its related factor, distinguishing it from the CH and F scales in all three cohorts.

In 2024, Dakin [40] conducted exploratory and confirmatory factor analyses to investigate the structure of various frequently referenced EB questionnaires. They described a four-factor model whereby Factor 1 encompassed food responsiveness, susceptibility to hunger, and disinhibition, while satiety responsiveness was negatively loaded onto this factor. These scales describe the automatic or implicit motivation to eat, which is, in principle, consistent with the CH scale of the NZ-EBQ. The second factor in their analysis pertained to restricted eating, characterized by cognitive reasoning and deliberate decision-making processes that influence the food intake. These reflective processes controlling food intake are influenced by emotional states, social norms, habit formation, and the starting BMI [41]. Furthermore, the meaning of “restraint” is still uncertain, namely whether it relates to actively restricting one’s intake or the motivation or success of such an attempt. Therefore, restraint may be heterogeneous and vary across dieters who are successful or unsuccessful in maintaining a lower weight [42]. As a result of this lack of clarity, inconsistent findings, and lack of documented associations with physiological processes, restrained eating was not included in the NZ-EBQ. The third factor in their analysis was “negative emotional eating”, which encompasses emotional overeating, eating triggered by negative emotions, and disinhibition. Conversely, emotional undereating had an inverse loading effect on this factor. This factor is consistent with EE in the NZ-EBQ, where all the scale items are related to negative emotions. Factor 4 of their model, ‘homeostatic eating’, refers to the capacity to attentively perceive and respond to the body’s internal signals and hunger cues, necessitating conscious awareness of bodily sensations. The level of interoceptive awareness may vary significantly among a broad range of individuals; hence, we did not include items that directly relate to this factor.

Regarding the association between BMI and EB traits, which describe potential drivers of increased food intake, we compared the findings of the NZ-EBQ with those of Dakin and colleagues [43], who evaluated several EB traits and their association with energy intake (EI) and BMI in a large meta-analysis. They showed that EB traits, such as increased susceptibility to hunger, reduced satiety responsiveness, and increased emotional eating, were positively correlated with EI and BMI. However, EB traits such as food addiction, intuitive eating, mindful eating, restraint, hedonistic hunger, and external eating revealed conflicting results regarding EI and BMI. All three subscales of the NZ-EBQ showed significant positive correlations with BMI, and there are theoretical and conceptual similarities between increased ‘susceptibility to hunger’ and our scale of CH; ‘satiety responsiveness’ and our scale of F; and ‘emotional eating’ and our scale of EE.

Regarding the internal consistency of the NZ-EBQ, we evaluated the test-retest reliability in Cohort 2 and had previously assessed the test-retest reliability in Cohort 0. The results showed moderate to good reliability, which was better in Cohort 2 compared with the initial cohort. The EE scale showed superior reliability in all cohorts, as did the shorter, less ambiguous items. This finding aligns with several observations [30,36] that showed that emotional eating can be recognized by a person consistently and to a relatively high degree. The rationale behind the NZ-EBQ aligns with the concept of actionable EB traits, as described by Acosta [15], using EB traits or phenotypes to achieve superior responsiveness to treatment and reduce interindividual response variation. The proposed classification system of the NZ-EBQ describes three EB traits that align with the phenotypes described by Acosta, who identified four actionable phenotypes: abnormal satiation, abnormal postprandial satiety (duration of fullness), and emotional eating. He also described abnormal resting expenditure that cannot be measured using a questionnaire.

We observed different percentages of individuals with EE, CH, and F in the three cohorts (Table S2), consistent with the fact that the cohorts were intentionally chosen from diverse backgrounds. There were more participants with EE in Cohorts 1 and 2 than in the initial cohort; however, in all three cohorts, female participants were more emotional eaters than other EB types. Emotional eating is a recognized characteristic that connects depression and weight gain, especially in females [44]. Additionally, it serves as an indicator of elevated BMI in patients diagnosed with Major Depressive Disorder (MDD), who frequently demonstrate an increased propensity for engaging in excessive eating as a response to negative emotions [31]. In addition, Mills and colleagues [45] stated that EE behavior is more common in females and confirmed that obese females exhibit both higher EE and greater hunger.

Interestingly, approximately the same proportion of participants could not be assigned to either of the three cohorts (Cohort 0:9.6%; Cohort 1:12.3%, Cohort 2:13.0%), indicating that the categorization system was relatively stable for unassignable participants. The absolute numbers of unassignable participants align with the findings of Acosta [6], who showed that 15% of the participants were unclassifiable using his method. As previously mentioned, we asked people to categorize themselves into different EB traits, allowing for five options with recognizable terminology. When comparing individuals’ self-categorized EB with the trait assigned in the NZ-EBQ, emotional eating was the most recognized trait in all three cohorts. The analysis found a substantial overlap between CH and F, and the test-retest reliability was low, consistent with the findings from the initial cohort. This observation supports the use of NZ-EBQ instead of self-categorizing EB, which is insufficient and not recommended.

**Figure 3:**
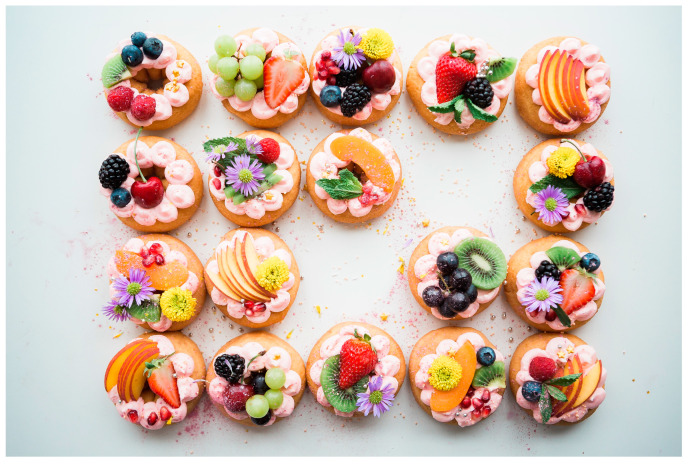
Attention checker item one. Instruction for participants: Please click on the cookie you would like to eat right now. Only one choice is allowed.

Consistent with the initial study, image-based questions using the concept of vividness of imagery and mental imagery affecting food cravings were added to alleviate questionnaire fatigue. In addition, two attention checkers were included based on the concept of instructed response items [21]. The image-based questions showed no significant associations with the EB classification and did not load onto the three EB scales; hence, they were not included in the analysis, and we eliminated them from the final tool. Nonetheless, two attention checkers (Figure 3) were included in the final tool to increase concentration and attention, and reduce missing answers near the end of the questionnaire.

However, this tool has several limitations. (1) As documented previously, every individual has several EB traits that co-exist, determine energy intake, and impact body weight. We observed that several individuals had more than one dominant EB trait. Since appetite is a complex sensation that includes multiple brain regions and peripheral signals with inter-system integration and an anatomical overlap between neural circuits regulating feeding, it is understandable that there will be limitations in identifying distinct behavioral traits with a questionnaire-based tool. Therefore, the NZ-EBQ must be integrated with other assessments to select the most suitable interventions. (2) The evaluation of EB traits through questionnaires can be affected by the inherent limitations of human memory, recall bias, and depression [10]. Such limitations in the approach are reflected in the moderate test-retest reliability observed in Cohorts 0 and 2. (3) In addition, many situational factors, such as being satiated before using the instrument, feeling rushed, or being interrupted while using the tool may have influenced the results. The strength of this study is the systematic approach to validate the NZ-EBQ in three independent cohorts, while the initial validation cohort had 73.8% of participants with type 2 diabetes, Cohorts 1 and 2 had 12.3% and 16.2%, respectively. During this process, we excluded one item with cross-factor loading and confirmed that the model was more robust after removing this item. The NZ-EBQ had good internal consistency across different ethnicities and education levels, as well as with different starting body weights, intentions to lose weight, and use of weight management medications.

In the future, it will be essential to determine whether the NZ-EBQ is informative in clinical practice for improved treatment selection to achieve enhanced weight loss outcomes compared with current best practices, including clinical judgment and empirical decisions. Possible applications may include selecting interventions such as lifestyle, medication, or metabolic surgery in conjunction with various clinical tools, such as semi-structured interviews, ad-libitum meal tests to measure satiation, and gastric emptying studies to measure postprandial satiety [7,46]. Furthermore, the NZ-EBQ can be used to monitor treatment progress and changes in individual EB during or after an intervention using repeated measures. Since EB often changes before weight loss is achieved [47], having a tool that identifies changes early on could help clinicians adjust medication choices during the initial periods of treatment, reducing costs and avoiding inefficient treatments.

## 5. Conclusions

The NZ-EBQ, validated in three independent cohorts derived from different population groups and refined in this study by excluding items with insufficient factor loading, is intended to support clinicians in identifying actionable eating behavior traits, which are defined as unique traits that can be targeted with specific tailored treatments, including weight loss medication and dietary interventions. This study showed that the factor structure and item loading are consistent across a wide range of clinical cohorts, including those on weight loss medications. Studies testing its applicability as a clinical assessment tool are warranted.

## Figures and Tables

**Table 1:** Demographics, weight, and weight loss characteristics of participants in two cohorts compared to the initial cohort.

	Initial Cohort (n = 729)	Cohort 1 (n = 81)	Cohort 2 (n = 215)
**Age category**			
18–30 years	12 (1.6%)	14 (17.3%)	21 (9.8%)
31–45 years	98 (13.4%)	17 (21.0%)	88 (40.9%)
46–60 years	244 (33.5%)	33 (40.7%)	76 (35.3%)
61–75 years	300 (41.1%)	14 (17.3%)	28 (13.0%)
76 years or older	75 (10.3%)	3 (3.7%)	2 (0.9%)
**Gender**			
Male	415 (56.9%)	26 (32.1%)	48 (22.3%)
Female	313 (42.9%)	55 (67.9%)	164 (76.3%)
Non-binary	1 (0.1%)	0 (0.0%)	3 (1.4%)
**Highest education completed**			
Primary school	22 (3.0%)	1 (1.2%)	12 (5.6%)
Secondary school	410 (56.2%)	26 (32.1%)	127 (59.1%)
Bachelor degree	207 (28.4%)	43 (53.1%)	65 (30.2%)
Master or doctoral degree	90 (12.3%)	11 (13.6%)	11 (5.1%)
**Perception of current weight**			
Underweight	20 (2.7%)	0 (0.0%)	0 (0.0%)
Neither underweight nor overweight	214 (29.4%)	11 (13.6%)	12 (5.6%)
Overweight	370 (50.7%)	45 (55.6%)	48 (22.3%)
Very overweight	125 (17.1%)	25 (30.9%)	155 (72.1%)
**Want to lose weight**	556 (76.3%)	72 (88.9%)	209 (97.2%)
**Currently taking prescription weight loss medication**	15 (2.1%)	31 (38.3%)	19 (8.8%)
**Current weight (kg)**	Mean (95% CI)	Mean (95% CI)	Mean (95% CI)
	97.2 (95.2–99.1)	102.0 (96.0–108.9)	139.3 (133.6–145.0)

**Table 2:** Differences in eating behavior classification of the initial cohort using the original model and the new model.

Classification of Eating Behavior	Original Model	New Model	% Change
Emotional eater	190 (26.1%)	192 (26.3%)	+1.0%
Feaster	162 (22.2%)	155 (21.2%)	−4.3%
Constant hunger	322 (44.2%)	312 (42.8%)	−3.1%
Unassigned	55 (7.5%)	70 (9.6%)	+27.3%

The original model refers to the model derived from the validation cohort [13] and includes item 7: “Even though I know better, I continue eating in the same way when my eating causes me emotional or social problems”. The new model represents the revised questionnaire and does not include item 7.

**Table 3:** Test-retest reliability of survey items among Cohort 2 using intraclass correlation coefficients (ICC).

No.	Item	ICC	95% CI
1	When I feel anxious, worried, or tense, I find myself eating.	0.79	0.63–0.88
2	I can get moody when I can’t have certain foods I crave, and I feel bad or empty if I deny myself having them.	0.60	0.36–0.77
3	When I feel lonely, I console myself by eating.	0.67	0.45–0.81
4	When I feel emotionally upset or when someone lets me down, I have a strong desire to reward myself with food.	0.90	0.82–0.95
5	I eat when I’m down.	0.84	0.71–0.91
6	When I feel sad, blue, or disappointed, I often overeat and/or have the desire to eat.	0.89	0.80–0.94
8	I have a desire to eat when things have gone wrong.	0.89	0.80–0.94
9	When you are going through a stressful or upsetting time what happens to your eating?	0.77	0.60–0.87
10	I know that I’m an emotional eater.	0.92	0.81–0.96
11	During a meal, I have no sense of being full and can’t stop until I’m really stuffed.	0.53	0.26–0.73
12	When I’m preparing food I cannot stop tasting and eating while preparing.	0.72	0.53–0.84
13	Are you able to stop eating whenever you want?	0.50	0.21–0.70
14	I can get hungry again in a few hours, even if I just had a large meal.	0.53	0.26–0.73
15	Do you eat large portion sizes?	0.50	0.22–0.71
16	When I’m hungry I lose control over how much I eat and find it hard to stop eating.	0.69	0.48–0.83
17	Do you feel a sensation of fullness during eating?	0.48	0.19–0.69
18	Once I start eating, I have trouble stopping.	0.68	0.47–0.82
19	How difficult would it be for you to stop eating halfway through a meal (not finish your plate)?	0.63	0.40–0.79
20	How difficult is it for you not to overeat at a large tasty meal?	0.78	0.61–0.88
21	I have cravings or urges to eat snacks after supper for before bedtime.	0.71	0.52–0.84
22	When I see a real delicacy, I often get so hungry that I have to eat right away.	0.62	0.38–0.78
23	Whenever I have a food craving, I keep on thinking about eating until I actually eat the food.	0.46	0.18–0.68
24	How often do you feel so hungry that you just have to eat something?	0.65	0.42–0.80
25	I am always hungry enough to eat at any time.	0.81	0.67–0.90
26	If I haven’t eaten for a while I find it very hard to resist my hunger.	0.81	0.67–0.90
27	I have a habit of eating too much at night and I’m not hungry in the morning.	0.69	0.48–0.82

## Data Availability

The anonymized data presented in this study are available on request to interested researchers from the corresponding author (OS) in order not compromise anonymity of the participants and breach of local data protection laws. Data can only be shared in accordance with the consent provided by the participants. The data set has been archived at Te Toka Tumai Auckland on a password-protected institutional intranet. Requests to access the datasets should be directed to Dr. O Schmiedel (oles@adhb.govt.nz).

## Supplementary Materials

The following supporting information can be downloaded at: https://www.mdpi.com/article/10.3390/nu17061049/s1, Table S1. Model fit statistics of the three-factor model with and without item 7 in the two cohorts (combined) compared to the initial cohort; Table S2. Comparison of eating behavior classification among the three cohorts using the new model; Table S3. Median scores and interquartile range of relevant factor scores for each eating behavior type classification by cohort; Table S4. Demographics of participants by eating behavior classification in Cohorts 1 and 2 (Combined); Table S5. Comparison of eating behavior classification in Phase I and Phase II among Cohort 2; Table S6. Comparison of assigned eating behavior type with self-assessed behavior among Cohorts 1 and 2 (Combined); Figure S1: Correlation between individual BMI and EB scores.

## Abbreviations

BES	Binge Eating Scale
CFI	Comparative Fit Index
CFA	Confirmatory Factor Analysis
CH	Constant Hunger
DEBQ	Dutch Eating Behavior Questionnaire
EB	Eating Behavior
EE	Emotional Eating
EFA	Exploratory Factor Analysis
F	Feasting
ICC	Intraclass Correlation Coefficients
KMO	Kaplan Meyer Olkin measure of sampling adequacy
NZ-EBQ	New Zealand Eating Behavior Questionnaire
RMSEA	Root-mean-square error of approximation
SRMR	Standardized root-mean-square residual
TFEQ	Three Factor Eating Questionnaire
TLI	Tucker-Lewis index
YFAS	Yale Food Addiction Scale

## References

[1] Blüher M. (2019). Obesity: Global epidemiology and pathogenesis. Nat. Rev. Endocrinol..

[2] Upadhyay J., Farr O., Perakakis N., Ghaly W., Mantzoros C. (2018). Obesity as a disease. Med. Clin. N. Am..

[3] MacLean P.S., Rothman A.J., Nicastro H.L., Czajkowski S.M., Agurs-Collins T., Courcoulas A.P., Ryan D.H., Bessesen D.H., Loria C.M. (2018). The accumulating data to optimally predict obesity treatment (ADOPT) core measures project: Rationale and approach. Obesity.

[4] Roberts C.A., Christiansen P., Halford J.C.G. (2017). Tailoring pharmacotherapy to specific eating behaviours in obesity: Can recommendations for personalised therapy be made from the current data?. Acta Diabetol..

[5] Cawley J., Biener A., Meyerhoefer C., Ding Y., Zvenyach T., Smolarz B.G., Ramasamy A. (2021). Direct medical costs of obesity in the united states and the most populous states. J. Manag. Care Spec. Pharm..

[6] Acosta A., Camilleri M., Abu Dayyeh B., Calderon G., Gonzalez D., McRae A., Rossini W., Singh S., Burton D., Clark M.M. (2021). Selection of antiobesity medications based on phenotypes enhances weight loss: A pragmatic trial in an obesity clinic. Obesity.

[7] Camilleri M., Acosta A. (2016). Gastrointestinal traits: Individualizing therapy for obesity with drugs and devices. Gastrointest. Endosc..

[8] Dakin C., Finlayson G., Stubbs R.J. (2024). Exploring the underlying psychological constructs of self-report eating behavior measurements: Toward a comprehensive framework. Psychol. Rev..

[9] Hopkins M., Beaulieu K., Gibbons C., Halford J.C.G., Blundell J., Stubbs J., Finlayson G. The Control of Food Intake in Humans. https://www.endotext.org.

[10] Gibbons C., Hopkins M., Beaulieu K., Oustric P., Blundell J.E. (2019). Issues in measuring and interpreting human appetite (satiety/satiation) and its contribution to obesity. Curr. Obes. Rep..

[11] Dakin C., Finlayson G., Stubbs R.J. (2024). Investigating motivations to eat: Refining and validating a framework of eating behaviour traits in dieters and the general population. Appetite.

[12] Blundell J. (1991). Pharmacological approaches to appetite suppression. Trends Pharmacol. Sci..

[13] Blundell J., de Graaf C., Hulshof T., Jebb S., Livingstone B., Lluch A., Mela D., Salah S., Schuring E., Van Der Knaap H. (2010). Appetite control: Methodological aspects of the evaluation of foods. Obes. Rev..

[14] Schmiedel O., Ivey M., Liu A., Murphy R. (2023). The New Zealand eating behavior questionnaire—Validation study for a novel assessment tool to describe actionable eating behavior traits. Appetite.

[15] Acosta A., Camilleri M., Shin A., Vazquez-Roque M.I., Iturrino J., Burton D., O’Neill J., Eckert D., Zinsmeister A.R. (2015). Quantitative gastrointestinal and psychological traits associated with obesity and response to weight-loss therapy. Gastroenterology.

[16] Stunkard A.J., Messick S. (1985). The three-factor eating questionnaire to measure dietary restraint, disinhibition and hunger. J. Psychosom. Res..

[17] Karlsson J., Persson L., Sjöström L., Sullivan M. (2000). Psychometric properties and factor structure of the three-factor eating questionnaire (TFEQ) in obese men and women. results from the Swedish Obese Subjects (SOS) study. Int. J. Obes..

[18] Löffler A., Luck T., Then F.S., Sikorski C., Kovacs P., Böttcher Y., Breitfeld J., Tönjes A., Horstmann A., Löffler M. (2015). Eating behaviour in the general population: An analysis of the factor structure of the German version of the three-factor-eating-questionnaire (TFEQ) and its association with the body mass index. PLoS ONE.

[19] Anglé S., Engblom J., Eriksson T., Kautiainen S., Saha M.T., Lindfors P., Lehtinen M., Rimpelä A. (2009). Three factor eating questionnaire-R18 as a measure of cognitive restraint, uncontrolled eating and emotional eating in a sample of young Finnish females. Int. J. Behav. Nutr. Phys. Act.

[20] de Lauzon B., Romon M., Deschamps V., Lafay L., Borys J.M., Karlsson J., Ducimetière P., Charles M.A. (2004). The three-factor eating questionnaire-R18 is able to distinguish among different eating patterns in a general population. J. Nutr..

[21] Gummer T., Roßmann J., Silber H. (2021). Using instructed response items as attention checks in web surveys: Properties and implementation. Sociol. Methods Res..

[22] Jensterle M., Rizzo M., Haluzík M., Janež A. (2022). Efficacy of GLP-1 RA approved for weight management in patients with or without diabetes: A narrative review. Adv. Ther..

[23] Davies M.J., Bergenstal R., Bode B., Kushner R.F., Lewin A., Skjøth T.V., Andreasen A.H., Jensen C.B., DeFronzo R.A. (2015). Efficacy of liraglutide for weight loss among patients with type 2 diabetes: The SCALE diabetes randomized clinical trial. JAMA J. Am. Med. Assoc..

[24] Comrey A.L., Lee H.B. (1992). A First Course in Factor Analysis.

[25] Landis J.R., Koch G.G. (1977). The measurement of observer agreement for categorical data. Biometrics.

[26] Koo T.K., Li M.Y. (2016). A guideline of selecting and reporting intraclass correlation coefficients for reliability research. J. Chiropr. Med..

[27] Schulte E.M., Gearhardt A.N. (2017). Development of the modified yale food addiction scale version 2.0. Eur. Eat. Disord. Rev..

[28] Blundell J.E., Lawton C.L., Hill A.J. (1993). Mechanisms of appetite control and their abnormalities in obese patients. Horm. Res..

[29] Hunot C., Fildes A., Croker H., Llewellyn C.H., Wardle J., Beeken R.J. (2016). Appetitive traits and relationships with BMI in adults: Development of the adult eating behaviour questionnaire. Appetite.

[30] van Strien T. (2018). Causes of emotional eating and matched treatment of obesity. Curr. Diab. Rep..

[31] Konttinen H., van Strien T., Männistö S., Jousilahti P., Haukkala A. (2019). Depression, emotional eating and long-term weight changes: A population-based prospective study. Int. J. Behav. Nutr. Phys. Act..

[32] Van Strien T., Frijters J.E.R., Bergers G.P.A., Defares P.B. (1986). The Dutch Eating Behavior Questionnaire (DEBQ) for assessment of restrained, emotional, and external eating behavior. Int. J. Eat. Disord..

[33] Nagl M., Hilbert A., de Zwaan M., Braehler E., Kersting A. (2016). The German version of the dutch eating behavior questionnaire: Psychometric properties, measurement invariance, and population-based norms. PLoS ONE.

[34] Barrada J.R., van Strien T., Cebolla A. (2016). Internal structure and measurement invariance of the Dutch eating behavior questionnaire (DEBQ) in a (nearly) representative Dutch community sample. Eur. Eat. Disord. Rev..

[35] Acosta A., Cifuentes L., Anazco D., O’Connor T., Hurtado M., Ghusn W., Campos A., Fansa S., McRae A., Madhusudhan S. (2024). Unraveling the variability of human satiation: Implications for precision obesity management. Res. Sq..

[36] Caroleo M., Primerano A., Rania M., Aloi M., Pugliese V., Magliocco F., Fazia G., Filippo A., Sinopoli F., Ricchio M. (2018). A real world study on the genetic, cognitive and psychopathological differences of obese patients clustered according to eating behaviours. Eur. Psychiatry.

[37] Gormally J., Black S., Daston S., Rardin D. (1982). The assessment of binge eating severity among obese persons. Addict. Behav..

[38] Beck A.T., Ward C.H., Mendelson M., Mock J., Erbaugh J. (1961). An inventory for measuring depression. Arch. Gen. Psychiatry.

[39] Segura-García C., Aloi M., Rania M., Ciambrone P., Palmieri A., Pugliese V., Moruno A.J.R., De Fazio P. (2015). Ability of EDI-2 and EDI-3 to correctly identify patients and subjects at risk for eating disorders. Eat. Behav. Int. J..

[40] Dakin C., Finlayson G., Stubbs R.J. (2024). Can eating behaviour traits be explained by underlying, latent factors? An exploratory and confirmatory factor analysis. Appetite.

[41] Bryant E.J., Rehman J., Pepper L.B., Walters E.R. (2019). Obesity and eating disturbance: The role of TFEQ restraint and disinhibition. Curr. Obes. Rep..

[42] Dakin C.A., Finlayson G., Horgan G., Palmeira A.L., Heitmann B.L., Larsen S.C., Sniehotta F.F., Stubbs R.J. (2023). Exploratory analysis of reflective, reactive, and homeostatic eating behaviour traits on weight change during the 18-month NoHoW weight maintenance trial. Appetite.

[43] Dakin C., Beaulieu K., Hopkins M., Gibbons C., Finlayson G., Stubbs R.J. (2023). Do eating behavior traits predict energy intake and body mass index? A systematic review and meta-analysis. Obes. Rev..

[44] Koning E., Vorstman J., McIntyre R.S., Brietzke E. (2022). Characterizing eating behavioral phenotypes in mood disorders: A narrative review. Psychol. Med..

[45] Mills J.G., Thomas S.J., Larkin T.A., Pai N.B., Deng C. (2018). Problematic eating behaviours, changes in appetite, and weight gain in major depressive disorder: The role of leptin. J. Affect. Disord..

[46] Chial H.J., Camilleri C., Delgado-Aros S., Burton D., Thomforde G., Ferber I., Camilleri M. (2002). A nutrient drink test to assess maximum tolerated volume and postprandial symptoms: Effects of gender, body mass index and age in health. Neurogastroenterol. Motil..

[47] Bettadapura S., Dowling K., Jablon K., Al-Humadi A.W., le Roux C.W. (2024). Changes in food preferences and ingestive behaviors after glucagon-like peptide-1 analog treatment: Techniques and opportunities. Int. J. Obes..

