# Supplementary material for: Broader Validation of New Zealand Eating Behavior Questionnaire as Clinical Assessment Tool to Identify Actionable Eating Behavior Traits
Source: Nutrients. 2025 Mar 17;17(6):1049. doi: 10.3390/nu17061049 (PMC11945943; doi:10.3390/nu17061049)
Supplement: Supplementary file 1 [file nutrients-17-01049-s001.zip › nutrients-3499354-supplementary.pdf]

nutrients

MDPI

Supplementary Materials:

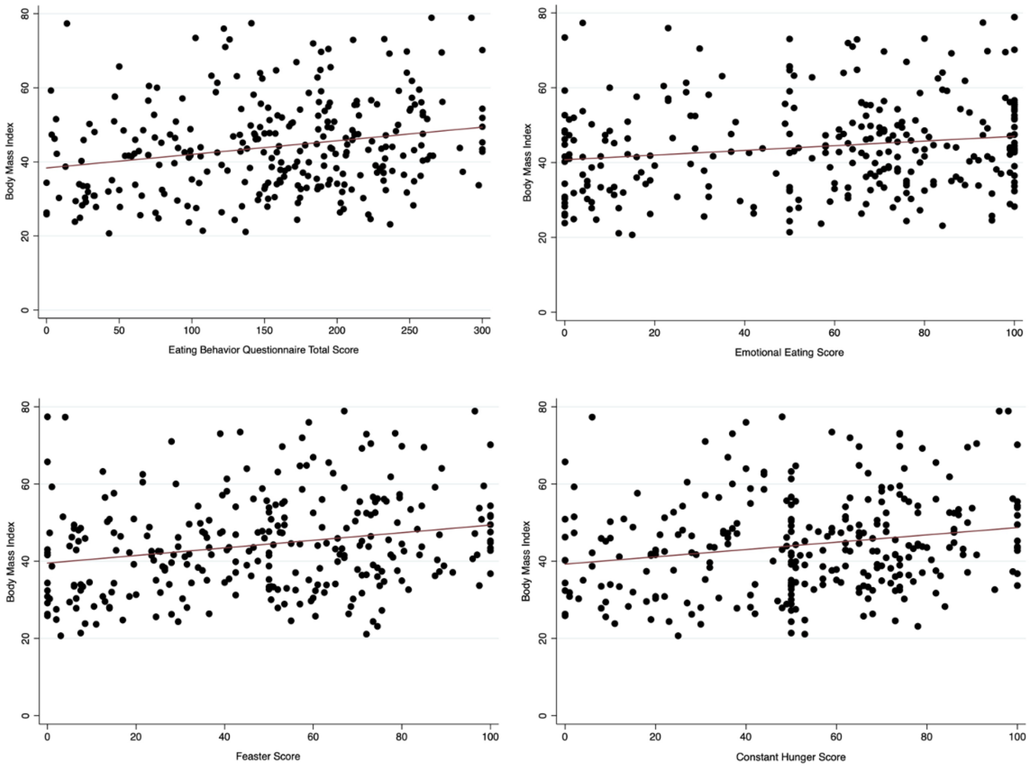

Figure S1: *Correlation between total and individual BMI and EB scores*

Participants total and eating behavior specific scores on the NZ-EBQ (0–100) compared to Body Mass Index (kg/m2).

Table S1. *Model Fit Statistics of Three Factor Model With and Without Item 7 In Two Cohorts (Combined) Compared to Initial Cohort*

	Cohort 0		Cohorts 1 and 2	
	**With**	**Without**	**With**	**Without**
Model fit statistics				
Comparative fit index (CFI)	0.909	0.900	0.911	0.911
Tucker-Lewis index (TLI)	0.901	0.891	0.903	0.903
Standardized root-mean-square residual (SRMR)	0.060	0.063	0.053	0.054
Root-mean-square error of approximation (RMSEA)	0.072	0.076	0.085	0.086
Overall R^2	0.998	0.998	0.998	0.998
Emotional eater factor α	0.95	0.94	0.97	0.97

Table legend:

Table S2. Comparison of eating behaviour classification among three cohorts using new mode.

Classification	Cohort 0	Cohort 1	Cohort 2	Overall
Emotional eater	192 (26.3%)	31 (38.3%)	96 (44.6%)	219 (31.1%)
Feaster	155 (21.3%)	18 (22.2%)	37 (17.2%)	210 (20.5%)
Constant hunger	312 (42.8%)	22 (27.2%)	54 (25.1%)	388 (37.8%)
Unassigned	70 (9.6%)	10 (12.3%)	28 (13.0%)	108 (10.5%)

Table legend: Pearson's chi-square test: $\chi^2 = 39.3$; $p = 0.000$.

Table S3. Median scores and interquartile range of relevant factor scores for each eating behaviour type classification by cohort.

Classification	Cohort 0	Cohort 1	Cohort 2
Emotional eater	68.00 (56.50 - 85.00)	70.00 (55.00 - 79.00)$^{\varphi}$	79.00 (68.5 - 95.00)$^{\delta}$
Feaster	33.50 (15.00 - 56.00)	68.50 (50.00 - 75.50)*	69.50 (48.50 - 87.50)$^{\delta}$
Constant hunger	50.00 (29.00 - 63.00)	50.00 (19.00 - 69.00)	66.00 (37.00 - 74.00)$^{\delta}$

Table legend: *P value < .05 for comparison between Cohort 0 and 1; δ P value < .05 for comparison between Cohort 0 and 2 ; φ P value < .05 for comparison between Cohort 1 and 2.

Table S4. Demographics of Participants By Eating Behaviour Classification in Cohorts 1 and 2 (Combined).

	Emotional Eater (n = 127)	Feaster (n = 55)	Constant Hunger (n = 76)	P value
**Diabetes diagnosis**				.26
No diabetes	79 (65.8%)	32 (64.0%)	38 (54.3%)	
Pre-diabetes	20 (16.7%)	11 (22.0%)	12 (17.1%)	
Type I	1 (0.8%)	1 (2.0%)	4 (5.7%)	
Type II	20 (16.7%)	6 (12.0%)	16 (22.9%)	
**Age category**				.77
18-30 years	13 (10.2%)	5 (9.1%)	12 (15.8%)	
31-45 years	45 (35.4%)	15 (27.3%)	27 (35.5%)	
46-60 years	45 (35.4%)	25 (45.4%)	27 (35.5%)	
61-75 years	22 (17.3%)	9 (16.4%)	9 (11.8%)	
76 years or older	2 (1.6%)	1 (1.8%)	1 (1.3%)	
**Gender**				.000
Male	18 (14.4%)	23 (41.8%)	27 (35.5%)	
Female	107 (85.6%)	32 (58.2%)	49 (64.5%)	
**Ethnicity**				.57
NZ European or Other European	79 (64.2%)	33 (62.3%)	35 (49.3%)	
Maori	14 (11.4%)	6 (11.3%)	10 (14.1%)	
Asian	8 (6.5%)	3 (5.7%)	7 (9.9%)	
Pacific Islander	22 (17.9%)	11 (20.8%)	19 (26.8%)	
**Highest education completed**				.15
Primary school	1 (0.8%)	4 (7.3%)	5 (6.6%)	
Secondary school	69 (54.3%)	26 (47.3%)	36 (47.4%)	
Bachelor degree	47 (37.0%)	19 (34.5%)	31 (40.8%)	
Master or doctoral degree	10 (7.9%)	6 (10.9%)	4 (5.3%)	
**Perception of current weight**				.18
Neither underweight nor overweight	7 (5.5%)	2 (3.6%)	9 (11.8%)	
Overweight	42 (33.1%)	14 (25.4%)	27 (35.5%)	
Very overweight	78 (61.4%)	39 (70.9%)	40 (52.6%)	
**Want to lose weight**	122 (96.1%)	54 (98.2%)	69 (90.8%)	.16

**Ever had weight loss surgery**	23 (18.1%)	3 (5.4%)	11 (14.5%)	.07
**Currently taking prescription weight loss medication**	25 (19.7%)	7 (12.7%)	9 (11.8%)	.26
	Mean (SD 95% CI)	Mean (SD 95% CI)	Mean (SD 95% CI)	
**Current weight (kg)**	126.2 (119.1-133.4)	136.4 (127.0-145.8)	128.1 (118.6-137.7)	.29

Table S5. Comparison of eating behaviour classification in phase I and phase II *.among Cohort 2*

			**Phase II classification**
**Phase I classification**	**Emotional Eater**	Feaster	Constant Hunger
Emotional Eater	15 (83.3%)	1 (5.6%)	2 (11.1%)
Feaster	2 (22.2%)	6 (66.7%)	1 (11.1%)
Constant Hunger	1 (14.3%)	1 (14.3%)	5 (71.43%)

Table S6. Comparison of Assigned Eating Behaviour Type with Self-Assessed Behaviour among Cohorts 1 and 2 (Combined).

		**Classification using scoring strategy**		
**Self-Assessed Eating Behavior**	**N**	**Emotional eater**	Feaster	Constant hunger
Binge eater	23 (8.9%)	16 (69.6%)	2 (8.7%)	5 (21.7%)
Emotional eater	69 (26.7%)	56 (81.2%)	7 (10.1%)	6 (8.7%)
Grazer or constant craving	50 (19.4%)	20 (40.0%)	8 (16.0%)	22 (44.0%)
Hedonistic eater	25 (9.7%)	14 (56.0%)	3 (12.0%)	8 (32.0%)
Large portion sizes but fewer meals	91 (35.3%)	21 (23.1%)	35 (38.5%)	35 (38.5%)
